# Supplementary material for: Married women’s decision-making autonomy in the household and maternal and neonatal healthcare utilization and associated factors in Debretabor, northwest Ethiopia
Source: PLoS One. 2021 Sep 27;16(9):e0255021. doi: 10.1371/journal.pone.0255021 (PMC8476028; doi:10.1371/journal.pone.0255021)
Supplement: S1 File — (DOCX) [file pone.0255021.s001.docx]

Annex 1: English version of the questionnaire

Part I: Socio-demographic characteristics

| N.O | Questionnaire | Alternative choice for response | Skip code |
| --- | --- | --- | --- |
| 101 | How old are you? | (In years) |  |
| 102 | What is your educational level? | 1. Can’t read and write  2. Can read and write  3. Primary  4. Secondary  5. Diploma and above |  |
| 103 | What is your occupation? | 1.House wife  2. Merchant  3. Government employee  4. self employed  5. Student  6. Others specify…………………………. |  |
| 104 | What is your religion? | 1. Orthodox Christian  2. Muslim  3. Protestant  4. Catholic  5. Other specify…………………………. |  |
| 105 | How much is the average monthly income of the family? | (In Ethiopian birr) |  |
| 106 | How many persons are living at home (family size)? |  |  |
| 109 | What is your husband’s educational level? | 1. Can’t read and write  2. Can read and write  3. Primary  4. Secondary  5. Diploma and above |  |
| 110 | What is your husband’s occupation? | 1. Daily labor  2. Merchant  3. Government employee  4. self employed  5. Student  6. Others specify…………………………. |  |
| 111 | Have you ever read newspapers? | 1. Yes 2. No | If yes got to Q 111 |
| 112 | If yes, how often did you read? | 1. Every day 2. One times per week 3. Two times per week 4. Three and more |  |
| 113 | Have you ever heard radio? | 1. Yes 2. No | If yes got to Q 113 |
| 114 | If yes, how often did you heard? | 1. Every day 2. One times per week 3. Two times per week 4. Three and more |  |
| 115 | Have you ever watched a television? | 1. Yes 2. No | If yes got to Q 115 |
| 116 | If yes, how often did you watched? | 1. Every day 2. One times per week 3. two times per week 4. Three and more |  |

Part II: Reproductive and maternity health services related characteristics

| NO | Questioners | Alternative choices for response | Skip code |
| --- | --- | --- | --- |
| 201 | How many times did you give birth after 7 months (parity)? |  |  |
| 202 | Did you have ANC follow-up in your most recent pregnancy? | 1. Yes  2. No | If yes got to 203 |
| 203 | How many ANC visits did you had on your most recent pregnancy? | 1. Once  2. Twice  3. Three times  4. Four and above |  |
| 204 | Where did you deliver your most recent child? | 1. 1. At home 2. 2. At health post 3. 3. At health center   4. At government hospital  5. At private clinic/hospital  6. Other specify |  |
| 205 | Who assisted your most recent delivery? | 1. 1. Health professional 2. 2. Traditional birth attendants 3. 3. Family 4. 4. Other specify |  |
| 206 | Did you have PNC visit in your most recent child? | 1. 1. Yes 2. 2. No | If no go to 208 |
| 207 | How many PNC visits did you have? | 1. 1. One 2. 2. Two 3. 3. Three or more |  |
| 208 | Did health extension workers visited you after your recent delivery or during pregnancy? | 1. 1. Yes 2. 2. No |  |
| 209 | Did you have a history of neonatal death? | 1. 1. Yes   2. No |  |
| 215 | Husband/Partner involvement related questions on MNCH services | | |
| I | Did your husband go with you for ANC follow up at least once in your most recent pregnancy? | 1. Yes  2. No |  |
| II | Did your husband provide transport/gave money for transport during your recent pregnancy or delivery? | 1.Yes  2. No |  |
| III | Did your husband accompany to the hospital during labor for your recent delivery? | 1. Yes  2. No |  |
| IV | Did your husband discuss with health care providers during your recent pregnancy or delivery? | 1. Yes  2. No |  |
| V | Did your husband look after the child at home/stay with babies while you are outside home? | 1. Yes  2. No |  |
| VI | Did your husband bath newborn/infant while you are busy? | 1. Yes  2. No |  |
| VII | Did your husband buy clothes/other things for infants/neonates? | 1. Yes  2. No |  |
| VIII | Did your husband go with you for immunization services? | 1. Yes  2. No |  |
| IX | Did your husband assisted you while you breastfeed the newborn? | 1. Yes  2. No |  |
| 210 | How long does it take from your home to the nearby health facility on foot? | 1. 1. < 30 minute   2. ≥ 30 minute |  |

Part III: Mothers knowledge of neonatal danger signs

| No | Questionnaire | Alternative choice for response | Skip code |
| --- | --- | --- | --- |
| 301 | Have you ever heard about neonatal danger signs? | 1. 1.Yes 2. 2. No |  |
| 302 | List neonatal dangers sign that you knew? | 1. 1. Diarrhea 2. 2. High-grade fever 3. 3. Low temperature/cold body 4. 4. Fast breathing 5. 5.Sever chest in drawing/difficulty in breathing 6. 6. Yellowish discoloration of the body 7. 7. Unconsciousness 8. 8. Moves only when stimulated/lethargy 9. 9. Convulsion   10. Umbilical redness/draining pus  11. Eyes draining pus  12. Failure to suck  13. Others |  |

Part V: Question on health seeking practice of mothers

| No | Questionnaire | Alternative choice for response | Skip code |
| --- | --- | --- | --- |
| 401 | Did your baby experience one or more of the neonatal danger signs in the first 28 days after delivery? | 1. 1. Yes 2. 2. No |  |
| 402 | If yes, which type of neonatal danger sign was experienced to your baby? | 1. Diarrhea  2. High-grade fever  3. Low temperature/cold body  4. Fast breathing  5. Sever chest in drawing/difficulty of breathing  6. Yellowish discoloration  7. Unconsciousness/lethargy  8. Convulsion  9. Moves only when stimulated  10. Umbilical redness/draining pus  11. Eyes draining pus  12. Failure to suck  13. Others |  |
| 403 | What did you do for your sick baby? | 1. Take to the health post  2. Take to health center  3. Take to governmental hospital  4. Take to private clinic/hospital  5.Purchase drug from a pharmacy without prescription  6. Take to traditional healer  7. Giving home remedies  8. Take to spiritual healer/temple  9. Do nothing  10. Other specify |  |

Part V: Decision-making autonomy related questions

| No | Questionnaire | Alternative choice for response | Skip code |
| --- | --- | --- | --- |
| 501 | Who decisions about health care for yourself? | 1. Me alone 2. My husband alone 3. Both of us 4. Others specify ……………………… |  |
| 502 | Who decides on large household purchase or sell? | 1. Me alone 2. My husband alone 3. Both of us 4. Others specify ……………………… |  |
| 503 | Who decides on intrahousehold resource allocation/ daily household purchases? | 1. Me alone 2. My husband alone 3. Both of us 4. Others specify ……………………… |  |
| 504 | Who decides on where and when to seek medical care for sick newborns/children? | 1. Me alone 2. My husband alone 3. Both of us 4. Others specify ……………………… |  |
| 505 | Who decides on visits of family, friends or relatives? | 1. Me alone 2. My husband alone 3. Both of us 4. Others specify ……………………… |  |
| 506 | Who decides when to have an additional child? | 1. Me alone 2. My husband alone 3. Both of us 4. Others specify ……………………… |  |
| 507 | Who usually decides how your partner’s/husband earnings will be used? | 1. Me alone 2. My husband alone 3. Both of us 4. Others specify ……………………… |  |
| 508 | Who decides to go for ANC visit, PNC visit, where to deliver and infant immunization? | 1. Me alone  2. My husband alone  3. Both of us  4. Others specify ……………………… |  |
| 509 | Who usually decides what foods to be cooked each day? | 1.Me alone  2.My husband alone  3.Both of us  4. Others specify ……………………… |  |

#

Annex **2**: Amharic version of the questionnaire (የመጠይቁ አማርኛ ክፍል)

ክፍል አንድ: ማህበራዊ እና ኢኮኖሚያዊ ነክ ጥያቄዎች

| ቁ | መጠይቅ | የተሰጡ አማራጮች | ኮድ ይዝለሉ |
| --- | --- | --- | --- |
| 101 | እድሜዎ ስንት ነው |  |  |
| 102 | የትምህርት ደረጃዎ ስንት ነው | 1. ማንበብና መጻፍ የማይትችል  2. ማንበብና መጻፍ የምትችል  3. ከአንድ-ስምንተኛ ክፍል  4. ከዘጠኝ-አስራ ሁለት ክፍል  5. ዲፕሎማ እና ከዛ በላይ |  |
| 103 | ስራዎ ምንድነው | 1. የቤት እመቤት  2. የመግስት ሰራተኛ  3. የግል ተቀጣሪ  4. ነጋዴ  5. ተማሪ  6. ሌላ ካለ ይጠቀስ |  |
| 104 | ሀይማኖቶ ምንድነው | 1. ኦርቶዶክስ ክርስቲያን  2. ሙስሊም  3. ፕሮቴስታንት  4. ካቶሊክ  5. ሌላ ካለ ይጠቀስ |  |
| 105 | የቤተሰቡ አማካይ የወር ገቢ ስንት ነው |  |  |
| 106 | ቤት ውስጥ ያለ የቤተስብ ብዛት ስንት ነው |  |  |
| 109 | የባለቤቶ የትምህርት ደረጃ ስንት ነው | 1. ማንበብና መጻፍ የማይችል  2. ማንበብና መጻፍ የሚችል  3. ከአንድ-ስምንተኛ ክፍል  4. ከዘጠኝ-አስራ ሁለት ክፍል  5. ዲፕሎማ እና ከዛ በላይ |  |
| 110 | የባለቤቶ ስራ ምንድነው | 1. የቀን ሰራተኛ  2. የመግስት ሰራተኛ  3. የግል ተቀጣሪ  4. ነጋዴ  5. ተማሪ  6. ሌላ ካለ ይጠቀስ |  |
| 111 | ጋዜጣ አንብበዉ ያዉቃሉ | 1. አዎ  2. የለም |  |
| 112 | አዎ ከሆነ ምን ያክል ጊዜ ያነባሉ | 1. በየቀኑ 2. በሳምንት ጊዜ 3. በሳምንት 2 ጊዜና ከዚያ በላይ 4. ሌላ ካለ ይገለጽ |  |
| 113 | ሬድዮ አዳምጠዉ ያዉቃሉ | 1. አዎ  2. የለም |  |
| 114 | አዎ ከሆነ ምን ያክል ጊዜ ያዳምጣሉ | 1. በየቀኑ 2. በሳምንት ጊዜ 3. በሳምንት 2 ጊዜና ከዚያ በላይ 4. ሌላ ካለ ይገለጽ |  |
| 115 | ቴሌቬዠን ተመልክተው ያዉቃሉ | 1. አዎ  2. የለም |  |
| 116 | አዎ ከሆነ ምን ያክል ጊዜ ይመለከታሉ | 1. በየቀኑ  2. በሳምንት ጊዜ  3. በሳምንት 2 ጊዜና ከዚያ በላይ  4. ሌላ ካለ ይገለጽ |  |

ክፍል ሁለት: ከወሊድና እርግዝና ጋር የተያያዙ ጥያቄዎች

| ቁ. | መጠይቅ | የተሰጡ ኣመራጮች | ኮድ ይዝለሉ |
| --- | --- | --- | --- |
| 201 | ከ 7 ወር በኋላ ስንት ጊዜ ወልደዋል |  |  |
| 202 | የመጨረሻ ልጆን ሲያረግዙ የእርግዝና ክትትል ነበሮዎት? | 1. አዎ  2. የለም |  |
| 203 | ምን ያህል እርግዝና ክትትል ነበሮት? | 1. አንድ ጊዜ  2. ሁለት ጊዜ  3. ሦስት ጊዜ  4. አራት እና ከዚያ በላይ |  |
| 204 | የመጨረሻ ልጆን የት ነበር የወለዱት? | 1. ቤቴ  2. ጤና ኬላ  3. ጤና ጣቢያ  4. የመንግስት ሆስፒታል  5. የግል ክሊኒክ/ሆሰፒታል |  |
| 205 | የመጨረሻ ልጆን ማን ነበር ያዋለዶት? | 1. የጤና ባለሙያ  2. የልምድ አዋላጅ  3. ቤተሰብ  4. ሌላ |  |
| 206 | የመጨረሻ ልጆን ሲወልዱ የድህረ ወሊድ አገልግሎት አግኝተዋል? | 1. አዎ  2. የለም |  |
| 207 | ምን ያህል የድህረ ወሊድ ክትትል ነበሩዎት? | 1. አንድ  2. ሁለት  3. ሦስትና ከዚያ በላይ |  |
| 208 | የመጨረሻ ልጆን ከወለዱ በኋላ ወደ ጤና ኤክስቴንሽን ባለሞያዎች ሄደዋል/እነሱ ወደቤቶ መጥተው ጎብኝቶታል | 1. አዎ  2. የለም |  |
| 209 | ካሁን በፊት ህጻን ልጅ ሞቶቦት ያዉቃል? | 1. አዎ  2. የለም |  |
| 210 | ከባል ተሳትፎ ጋር የተያያዙ ጥያቄዎች | |  |
| I | በመጨረሻ እርግዝናዎ ወቅት ባለቤቶ ቢያንስ አንዴ ለእርግዝና ክትትል አብሮዎት ሂዶ ዉቃል? | 1. አዎ  2. የለም |  |
| II | ለእርግዝና ክትትል ወይንም ለሌላ የጤና ጉዳይ ወደ ጤና ተቋም ሲሄዱ ባለቤቶ የትራንስፖርት ገንዘብ ያዘጋጃል? | 1. አዎ  2. የለም |  |
| III | የመጨረሻ ልጅዎን ሲወልዱ ባለቤቶ ወደ ጤና ተቋም አድርሶታል/ከእርሶ ጋር አብሮ ወደ ጤና ተቋም ሄዷል? | 1. አዎ  2. የለም |  |
| IV | በመጨረሻ እርግዝናዋ ወይም የመጨረሻ ልጅዎን በሚወልዱበት ሰኣት ባለቤቶ ከጤና ሞያተኞች ጋር ተወያቷል? | 1. አዎ  2.የለም |  |
| V | የድህረ ወሊድ ክትትል ለማድርግ/ህፃናትን ለማስከተብ ወደጤና ተቋም ሲሄዱ ባለቤቶ አብሮት ሂዶ ያዉቃል? | 1. አዎ  2. የለም |  |
| VI | እርስዎ ከቤት ዉጭ በሚሆኑበት ሰኣት ባለቤቶ ህፃናትን ይጠብቃል/ያጫዉታል? | 1. አዎ  2. የለም |  |
| VII | ባለቤቶ ለህፃናት ልብስ / ሌላ አስፈላጊ ነገሮችን ይገዛል? | 1. አዎ  2. የለም |  |
| VIII | ባለቤቶ እርሶ ስራ ሲበዛቦት/ በራሱ ተነሳሽነት የህጻናትን ገላ ያጥባል? | 1. አዎ  2. የለም |  |
| IX | ባለቤቶ ጡት በሚያጠቡበት ሰኣት እንዴት ማጥባት እንዳለቦት ያግዞታል? | 1. አዎ  2. የለም |  |
| 211 | ከቤትዎ ወደ አቅራቢያ ጤና አገልግሎት ምን ያክል ጊዜ ይርቃል? | 1. <30 ደቂቃ  2. ≥ 30 ደቂቃ |  |

ክፍል ሦስት: ከእናቶች እውቀት ጋር የተገናኙ ጥያቄዎች

| ቁ. | መጠይቅ | የተሰጡ አማራጮች | ኮድ ይዝለሉ |
| --- | --- | --- | --- |
| 301 | ስለ ጨቅላ ህጻናት አደገኛ ምልክቶች ሰምተዉ ያዉቃሉ? | 1. አዎ  2. የለም |  |
| 302 | እሚያዉቋቸውን የጨቅላ ህጻናት አደገኛ ምልክቶች ይዘርዝሩልኝ | 1. ተቅማጥ  2. ከፍተኛ የሰውነት ትኩሳት  3. ዝቅተኛ ሙቀት ቀዝቃዛ ሰውነት  4. ፈጣን የሆነ አተነፋፈስ  5. አተነፋፈስ ችግር/ ሲተነፍስ ደረት ወደ ዉስጥ መግባት  6. የሰውነት ቢጫ መሆን  7. አዕምሮን መሳት/ራስን አለማወቅ  8. መልፈስፈስ/ሲነካካ ብቻ መንቀሳቀስ  9. የሰውነት መንዘፍዘፍ/መንቀትቀጥ  10. የእንብርት መቅላት / መግል መፍረጥ  11. በህጻኑ አይን መግል መፍረጥ  12. መጥባት አለመቻል |  |

ክፍል አራት- እናቶች ለታመሙ ህጻናት የሚያደርጉት ተግባር

| ቁ. | መጠይቅ | የተሰጡ አማራጮች | ኮድ ይዝለሉ |
| --- | --- | --- | --- |
| 401 | ከላይ አደገኛ የጨቅላ ህጻናት ምልክቶች ከተባሉት ዉስጥ በእርስዎ ልጅ ተከስቶ ያዉቃል? | 1. አዎ  2. የለም |  |
| 402 | መልሶ አዎ ከሆነ, በህጻኑ የትኞቹ አደገኛ የጨቅላ ህጻናት ምልክት ተከስቶበት ያዉቃል? | 1. ተቅማጥ  2. ከፍተኛ የሰውነት ትኩሳት  3. ዝቅተኛ ሙቀት / ቀዝቃዛ ሰውነት  4. ፈጣን የሆነ አተነፋፈስ  5. አተነፋፈስ ችግር/ ሲተነፍስ ደረት ወደ ዉስጥ መግባት  6. የሰውነት ቢጫ መሆን  7. አዕምሮን መሳት/ራስን አለማወቅ  8. መልፈስፈስ/ሲነካካ ብቻ መንቀሳቀስ  9. የሰውነት መንዘፍዘፍ/መንቀጥቀጥ  10. የእንብርት መቅላት / መግል መፍረጥ  11. በህጻኑ አይን መግል መፍረጥ  12. መጥባት አለመቻል |  |
| 403 | ልጅዎ ሲታመም ምን አደረጉ? | 1. ወደ ጤና ኬላ ወሰድኩ  2. ወደ ጤና ጣቢያ ወሰድኩ  3. ወደ መንግስት ሆስፒታል ወሰድኩ  4. ወደ ግል ክልኒክ/ሆስፒታል ወሰድኩ  5. ያለሃኪም ትዛዝ ከመዳሀኒት ቤት መዳኒት ገዛሁ  6. ከቤት ዉስጥ ያለ ነገር ሰጠሁት  8. ወደ ቤተ መቅደስ/መስጂድ ወሰድኩ  9. ወደ ባህል ሃኪም ወሰድኩ  10. ምንም አላደረኩም |  |

ክፍል አምስት: ከውሳኔ ጋር የተያያዙ ጥያቄዎች

| ቁ. | መጠይቅ | የተሰጡ አማራጮች |  |
| --- | --- | --- | --- |
| 501 | በምትታመሚበት ሰኣት ስላንቺ ጤና ማን ይወስናል (የት ሄደሽ መታከም እንዳለብሽ ማን ይወስናል)? | 1. እኔ ብቻየን  2. ባሌ ብቻውን  3. ሁለታችንም  4. ሌላ ካለ ይጠቀስ |  |
| 502 | በትልልቅ የቤት ዉስጥ ግዥና ሽያጮች ላይ ማን ይወስናል? | 1. እኔ ብቻየን  2. ባሌ ብቻውን  3. ሁለታችንም  4. ሌላ ካለ ይጠቀስ |  |
| 503 | ለቤት ውስጥ ፍጆታ የሚሆን ገንዘብ ማን ይበጅታል? | 1. እኔ ብቻየን  2. ባሌ ብቻውን  3. ሁለታችንም  4. ሌላ ካለ ይጠቀስ |  |
| 504 | ህጻናት ሲታመሙ የትና መቸ መታከም እንዳለባቸው ማን ይወስናል? | 1. እኔ ብቻየን  2. ባሌ ብቻውን  3. ሁለታችንም  4. ሌላ ካለ ይጠቀስ |  |
| 505 | ቤተሰብ ወይም ጓደኛ ለመጠየቅ ስትፈልጊ ማን ይወስናል? | 1. እኔ ብቻየን  2. ባሌ ብቻውን  3. ሁለታችንም  4. ሌላ ካለ ይጠቀስ |  |
| 506 | መቸ ተጨማሪ ልጅ መውለድ እንዳለባችሁ ማን ይወስናል? | 1. እኔ ብቻየን  2. ባሌ ብቻውን  3. ሁለታችንም  4. ሌላ ካለ ይጠቀስ |  |
| 507 | የባለቤቶ የወር ገቢ ለምን ስራ መዋል እንዳለበት ማን ይወስናል? | 1. እኔ ብቻየን  2. ባሌ ብቻውን  3. ሁለታችንም  4. ሌላ ካለ ይጠቀስ |  |
| 508 | ለእርግዝና ክትትል, የት ጤና ተቋም መውለድ እንዳለብዎት, የድህረ ወሊድ ክትትል ለማድርግ/ህፃናትን ለማስከተብ ማን ይወስናል? | 1. እኔ ብቻየን  2. ባሌ ብቻውን  3. ሁለታችንም  4. ሌላ ካለ ይጠቀስ |  |
| 509 | በየቀኑ ምን አይነት ምግብ መሰራት እንዳለበት ማን ይወስናል ? | 1. እኔ ብቻየን  2. ባሌ ብቻውን  3. ሁለታችንም  4. ሌላ ካለ ይጠቀስ |  |

አመሰግናለሁ!!
