# Supplementary material for: Married women’s decision-making autonomy in the household and maternal and neonatal healthcare utilization and associated factors in Debretabor, northwest Ethiopia
Source: PLoS One. 2021 Sep 27;16(9):e0255021. doi: 10.1371/journal.pone.0255021 (PMC8476028; doi:10.1371/journal.pone.0255021)
Supplement: Attachment — Submitted filename: PONE-D-20-25863_reviewerMaternal autonomy on Healthcare usage.pdf [file pone.0255021.s003.pdf]

PLOS ONE

Married Women’s Decision-Making Autonomy on Maternal and Neonatal Healthcare Utilization and Associated Factors in Debretabor, Northwest Ethiopia

--Manuscript Draft--

**Manuscript Number:**	PONE-D-20-25863
**Article Type:**	Research Article
**Full Title:**	Married Women’s Decision-Making Autonomy on Maternal and Neonatal Healthcare Utilization and Associated Factors in Debretabor, Northwest Ethiopia
**Short Title:**	Married Women’s Decision-Making Autonomy on Maternal and Neonatal Healthcare Utilization
**Corresponding Author:**	Azmeraw Ambachew Kebede, MSc<br>University of Gondar College of Medicine and Health Sciences<br>Gondar, Amhara regional State ETHIOPIA
**Keywords:**	Decision-making; Ethiopia; maternal and neonatal health
**Abstract:**	Background: Women’s decision-making autonomy is very crucial for the improvement of maternal, neonatal and child healthcare utilization. However, there is limited evidence on women’s decision-making autonomy on maternal and neonatal health in the study area. Therefore, this study assessed married women’s decision-making autonomy on maternal and neonatal healthcare utilization and associated factors in Debretabor, Northwest Ethiopia. The study is not clear whether MNHC is the only aspect of women autonomy that was considered<br>Methods: A community-based cross-sectional study was conducted from October 1st to 30th, 2019. A multi-stage sampling technique was used to select 730 married women. Data were collected using structured, pretested and interviewer-administered questionnaire. Data were entered into epi info 7 and analyzed by SPSS version 23. Multivariable logistic regression model was fitted to identify factors associated with women’s decision-making autonomy on maternal and neonatal healthcare utilization. Odds ratio with 95 % confidence interval was computed to determine the level of significance.<br>Result: Three-fourths (75.1%) of women had higher decision-making autonomy on their own health, neonatal health, and other social and economic aspects. Besides, the proportion of at least four antenatal visits, delivery at a health facility, postnatal checkup, knowledge of neonatal danger signs and appropriate health-seeking practices for sick newborns among autonomous women were 52.1%, 56.1%, 71.4%, 32%, and 80% respectively. Age greater than 35 years (AOR=2.08; 95%CI: 1.19, 3.62), monthly income of 5000 ETB and above (AOR=3.1; 95%CI: 1.36, 7.07), husband involvement (AOR=2.36; 95%CI: 1.55, 3.43) and knowledge of neonatal danger signs (AOR=2.11; 95%CI: 1.4, 3.2) were factors independently associated with women’s decision-making autonomy on maternal and neonatal healthcare utilization.<br>Conclusion: Our findings show that women’s decision-making autonomy on maternal and neonatal healthcare utilization was optimal. Increasing household income level through different means, promotion of husband’s involvement and increasing women’s knowledge of maternal and neonatal danger signs will have a great role for the improvement of women decision-making autonomy.
**Order of Authors:**	Azmeraw Ambachew Kebede, MSc<br>Getachew Azeze Eriku<br>Birhan Tsegaw Taye<br>Wagaye Fentahun Chanie
**Additional Information:**	
**Question**	**Response**
**Financial Disclosure**<br><br>Enter a financial disclosure statement that describes the sources of funding for the	No funding. We are from a low income country, Ethiopia.

work included in this submission. Review the submission guidelines for detailed requirements. View published research articles from *PLOS ONE* for specific examples.<br><br>This statement is required for submission and **will appear in the published article** if the submission is accepted. Please make sure it is accurate.<br><br>**Unfunded studies**<br>Enter: *The author(s) received no specific funding for this work.*<br><br>**Funded studies**<br>Enter a statement with the following details:<br>• Initials of the authors who received each award<br>• Grant numbers awarded to each author<br>• The full name of each funder<br>• URL of each funder website<br>• Did the sponsors or funders play any role in the study design, data collection and analysis, decision to publish, or preparation of the manuscript?<br>• **NO** - Include this sentence at the end of your statement: *The funders had no role in study design, data collection and analysis, decision to publish, or preparation of the manuscript.*<br>• **YES** - Specify the role(s) played.<br><br>* typeset	
**Competing Interests**<br><br>Use the instructions below to enter a competing interest statement for this submission. On behalf of all authors, disclose any competing interests that could be perceived to bias this work—acknowledging all financial support and any other relevant financial or non-financial competing interests.<br><br>This statement **will appear in the published article** if the submission is accepted. Please make sure it is accurate. View published research articles from *PLOS ONE* for specific examples.	We declare no competence of interest

**NO authors have competing interests**<br><br>Enter: *The authors have declared that no competing interests exist.*<br><br>**Authors with competing interests**<br><br>Enter competing interest details beginning with this statement:<br><br>*I have read the journal's policy and the authors of this manuscript have the following competing interests: [insert competing interests here]*<br><br>* typeset	
**Ethics Statement**<br><br>Enter an ethics statement for this submission. This statement is required if the study involved:<br><br>• Human participants<br>• Human specimens or tissue<br>• Vertebrate animals or cephalopods<br>• Vertebrate embryos or tissues<br>• Field research<br><br>Write "N/A" if the submission does not require an ethics statement.<br><br>General guidance is provided below. Consult the submission guidelines for detailed instructions. **Make sure that all information entered here is included in the Methods section of the manuscript.**	NA

**Format for specific study types**<br><br>**Human Subject Research (involving human participants and/or tissue)**<br>• Give the name of the institutional review board or ethics committee that approved the study<br>• Include the approval number and/or a statement indicating approval of this research<br>• Indicate the form of consent obtained (written/oral) or the reason that consent was not obtained (e.g. the data were analyzed anonymously)<br><br>**Animal Research (involving vertebrate animals, embryos or tissues)**<br>• Provide the name of the Institutional Animal Care and Use Committee (IACUC) or other relevant ethics board that reviewed the study protocol, and indicate whether they approved this research or granted a formal waiver of ethical approval<br>• Include an approval number if one was obtained<br>• If the study involved *non-human primates*, add *additional details* about animal welfare and steps taken to ameliorate suffering<br>• If anesthesia, euthanasia, or any kind of animal sacrifice is part of the study, include briefly which substances and/or methods were applied<br><br>**Field Research**<br><br>Include the following details if this study involves the collection of plant, animal, or other materials from a natural setting:<br>• Field permit number<br>• Name of the institution or relevant body that granted permission	
**Data Availability**<br><br>Authors are required to make all data underlying the findings described fully available, without restriction, and from the time of publication. PLOS allows rare exceptions to address legal and ethical concerns. See the PLOS Data Policy and FAQ for detailed information.	Yes - all data are fully available without restriction

A Data Availability Statement describing where the data can be found is required at submission. Your answers to this question constitute the Data Availability Statement and **will be published in the article**, if accepted.<br><br>**Important:** Stating 'data available on request from the author' is not sufficient. If your data are only available upon request, select 'No' for the first question and explain your exceptional situation in the text box.<br><br>Do the authors confirm that all data underlying the findings described in their manuscript are fully available without restriction?	
**Describe where the data may be found in full sentences. If you are copying our sample text, replace any instances of XXX with the appropriate details.**<br><br>• If the data are **held or will be held in a public repository**, include URLs, accession numbers or DOIs. If this information will only be available after acceptance, indicate this by ticking the box below. For example: *All XXX files are available from the XXX database (accession number(s) XXX, XXX.).*<br>• If the data are all contained **within the manuscript and/or Supporting Information files**, enter the following: *All relevant data are within the manuscript and its Supporting Information files.*<br>• If neither of these applies but you are able to provide **details of access elsewhere**, with or without limitations, please do so. For example:<br><br>*Data cannot be shared publicly because of [XXX]. Data are available from the XXX Institutional Data Access / Ethics Committee (contact via XXX) for researchers who meet the criteria for access to confidential data.*<br><br>*The data underlying the results presented in the study are available from (include the name of the third party*	All relevant data are within the manuscript and its Supporting Information files.

and contact information or URL).<br>• This text is appropriate if the data are owned by a third party and authors do not have permission to share the data.<br><br>* typeset	
Additional data availability information:	Tick here if your circumstances are not covered by the questions above and you need the journal's help to make your data available.

Powered by Editorial Manager® and ProduXion Manager® from Aries Systems Corporation

manuscript

Click here to access/download;Manuscript;manuscript on women auotnomy.docx

Type of manscript:

Original research article

Title:

Married Women's Decision-Making Autonomy on Maternal and Neonatal Healthcare Utilization and Associated Factors in Debretabor, Northwest Ethiopia

Name of authors:

Azmeraw Ambachew Kebede [1], Getachew Azeze Eriku [2], Birhan Tsegaw Taye [3], Wagaye Fentahun Chanie [4]

Affiliation:

[1] Department of Clinical Midwifery, School of Midwifery, College of Medicine and Health Sciences, University of Gondar, Gondar, Ethiopia; [2] Department of Physiotherapy, School of Medicine, College of Medicine and Health Sciences, University of Gondar, Gondar, Ethiopia; [3] Department of Midwifery, College of Medicine and Health Sciences, Debrebirhan University, Debrebirhan, Ethiopia; [4] UNFPA Supported Maternal Health Project Coordinator, College of Medicine and Health Sciences, University of Gondar, Gondar, Ethiopia

Correspondence: Azmeraw Ambachew Kebede

Po. Box 196 Gondar, Ethiopia

Tel: +251910880232

Email: azmuzwagholic@gmail.com

Abstract

Background: Women's decision-making autonomy is very crucial for the improvement of maternal, neonatal and child healthcare utilization. However, there is limited evidence on women's decision-making autonomy on maternal and neonatal health in the study area. Therefore, this study assessed married women's decision-making autonomy on maternal and neonatal healthcare utilization and associated factors in Debretabor, Northwest Ethiopia.

Methods: A community-based cross-sectional study was conducted from October 1st to 30th, 2019. A multi-stage sampling technique was used to select 730 married women. Data were collected using structured, pretested and interviewer-administered questionnaire. Data were entered into epi info 7 and analyzed by SPSS version 23. Multivariable logistic regression model was fitted to identify factors associated with women's decision-making autonomy on maternal and neonatal healthcare utilization. Odds ratio with 95 % confidence interval was computed to determine the level of significance.

Result: Three-fourths (75.1%) of women had higher decision-making autonomy on their own health, neonatal health, and other social and economic aspects. Besides, the proportion of at least four antenatal visits, delivery at a health facility, postnatal checkup, knowledge of neonatal danger signs and appropriate health-seeking practices for sick newborns among autonomous women were 52.1%, 56.1%, 71.4%, 32%, and 80% respectively.

Are these items not proxies women autonomy in this study

Age greater than 35 years (AOR=2.08; 95%CI: 1.19, 3.62), monthly income of 5000 ETB and above (AOR=3.1; 95%CI: 1.36, 7.07), husband involvement (AOR=2.36; 95%CI: 1.55, 3.43) and knowledge of neonatal danger signs (AOR=2.11; 95%CI: 1.4, 3.2) were factors independently associated with women's decision-making autonomy on maternal and neonatal healthcare utilization.

Conclusion: Our findings show that women's decision-making autonomy on maternal and neonatal healthcare utilization was optimal. Increasing household income level through different means, promotion of husband's involvement and increasing women's knowledge of maternal and neonatal danger signs will have a great role for the improvement of women decision-making autonomy.

Keywords: Decision-making, Ethiopia, maternal and neonatal health

Introduction

Women autonomy is defined as the ability of women to act independently on their particular health, children's health, freedom of movement and control over finance without requesting permission from somebody (1). Empowering women is very essential for any social and economic development of a country (2). Hence, women's autonomy undoubtedly contributes to many health advantages for both the mother and their children. However, there is a dearth of strong description of the concept and obtaining data on the individual, household and community level that shows all opportunities of women empowerment (3). Maternal and neonatal health provision needs a multi-sector approach which in turn requires a strong decision-making autonomy of women to reverse back the barriers at the household level (4).

Although maternal mortality has dropped by 2.9% every year between 2000-2017, there is still an estimated 295,000 maternal deaths and 2.6 million neonatal deaths in the year 2017 (5,6). Strong decision-making power of women is vital to drop this higher magnitude. Because, limited women's decision-making autonomy impedes maternal healthcare utilization such as antenatal care (ANC), postnatal care (PNC) and delivery at a facility (7). Besides, lower autonomy of women affect the socio-economic, emotional, fertility decision, contraceptive use and the sexual life of the women (8,9). Notably, decisions made at the household level affect not only the welfare of the individual but also the surrounding community even to the country level (10).

Studies show that the decision-making autonomy of women is low, specifically in developing countries; but scaling up the women's role in a decision leads to better uptake of healthcare access, poverty reduction and household economic growth (1,10,11). In developing countries, women play an important role in the beneficence of the family, but they essentially have seen as an ordinary homemakers (12). Literature in Ethiopia show that maternal and neonatal health coverage is low (13,14). Recent data revealed that 43%, 48% and 34% of women had four and above ANC visits, give birth at a health facility and postnatal check respectively (15). According to the 2016 Ethiopian demographic health survey (EDHS 2016), only 11-18% of women involved in making decisions alone and 66-68% together with their husband or partner (16). In addition, the neonatal mortality rate and the maternal mortality ratio of Ethiopia are 30 per 1000 live births (15) and 412 per 100,000 live births respectively (16).

So far, studies in Nigeria and in parts of Ethiopia including Bale zone, Ambo town, Southern Ethiopia and analysis from 2011 EDHS data revealed that women's decision-making autonomy was low; which was 38.9% (11), 41.4% (1), 55.6% (17), 58.4% (14) and 54% (18) respectively. Besides, studies from Iran, Nigeria and elsewhere in Ethiopia including Ambo town, Southern Ethiopia and Bale zone found that older maternal age, exposure to mass media, higher socioeconomic status, higher educational status, higher family size and knowledge of maternal and child health were positively associated with women's decision-making autonomy (1,8,11,14,17).

Improving maternal and neonatal health is one of the government concerns both nationally and globally, which comprises the third component of sustainable development goal (SDG) (19). Since improving the optimal health of both the mother and her neonate and typically decreasing the maternal and neonatal mortality as low as possible by assessing factors affecting maternal and neonatal healthcare utilization, specifically on autonomy of women will undoubtedly have a significant role for the achievement of the 3rd SDG. Even though a few researches were conducted on decision making autonomy of women, some of the published studies failed to address some variables like husband involvement in maternal, neonatal and child health (MNCH) services and knowledge of maternal and neonatal illness. Moreover, most of the published studies tried to correlate decision-making autonomy of women with maternal healthcare utilization only, leaving back what factors affect their decision-making autonomy.

In Ethiopia, there is significant burden of maternal and neonatal death due to low healthcare utilization (15,16). Promoting decision-making autonomy of women is the mainstay to increase maternal and neonatal healthcare utilization. Therefore, this study assessed married women's decision-making autonomy on maternal and neonatal healthcare utilization and associated factors in Debretabor, Northwest Ethiopia. There are two main objectives: i. how women's decision making autonomy affect MNHU.
ii. The factors that determine this decision making autonomy

Methods

Study design, setting and period

A community-based cross-sectional study was conducted from October 1st to 30th, 2019 in Debretabor, Northwest Ethiopia. The town is located at 665 km Northwest of Addis Ababa (the capital city of Ethiopia) and 103 km Northeast of Bahir Dar (the capital city of Amhara regional state) and it is the capital city of South Gondar Zone. Currently, the town has a total population of 63,563, of whom 31,863 (54.8 %) are female. An estimated 19,327 are women of the reproductive-age group. About 4317 (6.8%) are under five. Moreover, the town has one general hospital, three

State also how many are married, since that group is the unit of analysis if the data is available from your source

health centers, 6 Health posts and 6 private clinics serving the community (Debretabor administrative town report, unpublished data).

Study population

All married women who had an infant age of one year and below and residing for at least six months in the selected ‘kebeles were included in the study. Women who were seriously ill or mentally ill throughout the data collection period were excluded.

Sample size determination & sampling procedures

The sample size for this study was determined by using a single population proportion formula by considering the following assumptions :- the proportion of women’s-decision making autonomy on maternal and neonatal healthcare utilization 58.4% (14), 95% level of confidence and 5% margin of error.

$$n = \frac{Z\alpha/2)^2 p(1-p)}{(d)^2}$$

Where n= required sample sizes

α= level of significant

z= standard normal distribution curve value for 95% confidence level= 1.96

p= proportion of women’s decision-making autonomy on maternal and child healthcare utilization

d= margin of error

Therefore, $n=\frac{(1.96)^2 * 0.556(1-0.444)}{(0.05)2}$= 374

Finally, considering 10% non-response rate and a design effect of 2 (since multi-stage sampling was employed). Accordingly, the final calculated sample size was 748. A multi-stage sampling technique was employed to select the study subjects. In the first stage, three ‘kebeles’ were selected

by simple random sampling among the six ‘kebeles’, which is the smallest administrative unit in Ethiopia. The list of study participants was obtained from health extension workers and sampling frame was designed by numbering the list of women using the registration book. Finally, a total of 748 participants were selected by simple random sampling technique using a table of random generation. The calculated sample size was proportionally allocated to each ‘kebeles’.

Measurement

Women’s decision-making autonomy: For this study, it was composited to higher decision-making autonomy (which was coded as ‘‘1’’) and lower decision-making autonomy (which was coded as ‘‘0’’) and three response variables (2= for women who were able to decide individually, 1=for women who were able to decide together with her husband and 0 = otherwise). Accordingly, women who scored above the mean were considered highly autonomous whereas women who scored below the mean were less autonomous (1,2).

What is a response variable? this is confusing

The women were questioned “who in your household makes a decision about (1) Healthcare for yourself (2), Healthcare for newborns and/ or children (3) Visit of family or relatives (4) To have additional children (5) Utilization of maternal and child healthcare services like ANC, PNC and immunization (6) Large household purchases and consumptions (7) Intra-household resource allocation and purchases (8) Husband’s earning and (9) Cooking daily foods. The likely replies for each questions were women alone, together with her husband or husband only.

Justify the inclusion of these as components of MNHC

How about a situation where neither the husband or the woman make the decision on? I wonder that some of your respondents may fall into such category

What are the independent variables utilized in this analysis?

Operational definitions

Decision-making autonomy: Women who scored above the mean were considered highly autonomous whereas women who scored below the mean were less autonomous (1,2).

Good knowledge of NDS: Women who mentioned at least three neonatal danger signs among 12 neonatal danger signs (20).

What is NDS?

Poor knowledge NDS: Women who mentioned less than three neonatal danger signs among 12 neonatal danger signs (20)**.**

Appropriate health seeking practices: Women who sought care for neonatal danger signs from well-qualified health professionals in governmental and/or private health facilities (21)**.**

Inappropriate health seeking practices: Women who sought care for neonatal danger sign other than qualified health professionals like purchasing over the counter drugs, treating by home remedies, seeking care from temples and traditional healers (21).

Adequate ANC: Women who had four or more ANC visits (22).

Facility delivery: Delivery in public or private hospitals and/ or clinics (22).

Had PNC: Women who received at least one postnatal checkup (22).

Data collection tools and procedures

Data were collected using structured interviewer-administered questionnaire. Three diploma data collectors and three supervisors, who have bachelor of science in midwifery degree, trained about the interview technique collected and supervise the data.

Data quality controls

The questionnaire was first prepared in English and translated to the local Amharic language and back to English to keep the consistency of the questionnaire. Before the actual data collection pretest was done on 5% of the sample size at Adis Zemen town, Northwest Ethiopia to check response, language clarity, understanding of data collectors and supervisors about the questionnaire, and appropriateness of the questionnaire. The required modification was done before the actual data collection. During the actual data collection period, the questionnaire was checked for completeness daily by the supervisors and the principal investigator.

Data processing and analysis

Data were checked, coded and entered into EPI INFO version 7, and were exported to SPSS version 23 for analysis. Descriptive statistics were used to present participants' characteristics and decision-making autonomy of women. Binary logistic regression model was fitted to identify statistically significant explanatory variables and variables having a p-value of less or equal to 0.2 were included into the multivariable logistic regression model for controlling confounders. In multivariable logistic regression, a p-value of <0.05 with 95% CI for odds ratio was used to determine significance association. Model fitness was checked using Hosmer and Lemeshow goodness of fittest.

Ethics approval and consent to participate

The study was done in accordance with the Ethiopian Health Research Ethics Guideline and the declaration of Helsinki. Ethical clearance was obtained from the University of Gondar College of Medicine and Health Sciences ethical review committee. A formal letter of administrative approval was obtained from Debretabor town health office. A written informed consent was taken from each of the study participants after a clear explanation of the aim of the study.

Result

Socio-demographic characteristics of study participants

A total of 730 married women were included in this study. Eighteen women were excluded from the study due to their incomplete data, giving 98% response rate. The mean age of the participants was 30 years (SD ±5.86). Most of the study participants (97.9%) were Orthodox Christian by religion. Almost half (49.2%) of the participants had accomplished diploma and above education. Half (50.8%) of women were housewives by occupation. Regarding the occupation of the husband 406 (55.6%) were government employees and two-thirds (66.7%) of them had completed college and above education [**Table 1].**

Reproductive history and maternity healthcare service-related characteristics

From the total study participants, more than two-thirds (68.2%) of women had a parity of two to four. The majority of women (97.5%) had at least one ANC visit in their recent pregnancy, of whom, only 59.6% of women completed four ANC visits. Four-fifths (80.3%) of women gave birth at governmental health institutions for their recent delivery. Most (94%) of women had at least one postnatal visit for their most recent delivery [**Table 2].**

Women's decision-making autonomy at the household level

The overall decision-making autonomy of women was found to be 75.1% (95%CI: 72.1, 78.1).

From the measurement variable definition, what constitute overall DMA?

About 72.1% of women had the joint decision to visit the health facilities for their own health when they become sick. More than four-fifths (84.8%) of the study participants decide with their husbands to take sick newborns to the health facility. More than three-fourths (77.1%) and almost three-fourths (74.7%) of the participants had a joint decision with their husband for large household purchases and small intrahousehold resource allocation respectively [**Table 3].**

Decision-making status of women and MNCH service utilization

How was this variable derived and scored from your questionnaire?

The proportion of at least four ANC visits, delivery at a health facility, postnatal checkup, knowledge of neonatal danger signs and appropriate health-seeking practices for sick neonates among autonomous women were 52.1%, 56.1%, 71.4%, 32%, and 80% respectively.

I think you should be more specific about what you mean autonomous women: do you mean ' high autonomous women?'
This is one of your two main objectives. Did you attempt to investigate the significance of these proportion, rather include their respective confidence intervals, since this is a survey analysis of sample.

Factors associated with women's decision-making autonomy on maternal and neonatal healthcare utilization

You mentioned that your study differs from previous study because you added MNHU to the already known proxies for women autonomy index. Now,is this analysis based on just an aspect of the components of the proxies for women autonomy you itemized in ' measurement variable' section?

From the multivariable logistic regression analysis age of the women, monthly income of the family, knowledge of NDS and husband involvement in MNCH related activities had an association with women's decision-making autonomy.

times

Those women greater than 35 years were two (AOR=2.08; 95%CI: 1.19, 3.62) ~~times~~ more likely to had higher decision-making autonomy compared to their counterparts. Which category of age group is referred to here?

This can be a more acceptable term for the success in outcome

The odds of having higher decision-making autonomy among women who had a monthly income of 5000 ETB and above was three times higher compared to women who had a monthly income of less than 1200 ETB (AOR=3.10; 95% CI: 1.36, 7.07).

Similarly, those women who got husband involvement in MNCH related activities were 2.36 times (AOR=2.36; 95%CI: 1.60, 3.47) more likely to be autonomous compared to those women who didn't have husband support.

This study also revealed that those women who had adequate knowledge of neonatal dangers signs were two (AOR=2.11; 95%CI: 1.4, 3.2) times more likely to have had higher decision-making autonomy compared to women who had poor knowledge of newborn danger signs [**Table 4].**

Discussion

Women decision-making autonomy is very important for the wellbeing of the family, particularly for the improvement of maternal and neonatal health in resource-limited countries like Ethiopia. This study assessed married women's decision-making autonomy on maternal and neonatal healthcare utilization among women who had an infant of one year and below in Debretabor, Northwest Ethiopia. Three-fourths of women had higher decision-making autonomy on maternal and neonatal healthcare utilizations. Furthermore, this study found that the proportion of at least four ANC visits, delivery at a health facility, at least one postnatal checkup, good knowledge of neonatal danger signs and appropriate health-seeking practice for sick newborns among autonomous women was 52.1%, 56.1%, 71.4%, 32% and 80% respectively.

We found that the overall women's decision-making autonomy on maternal and neonatal healthcare utilization was 75.1% (95%CI: 72.1, 78.1), which was higher than previous studies conducted in Nigeria (11) and in parts of Ethiopia including Bale zone (1), Ambo town (17), Southern Ethiopia (14) and analysis from EDHS 2011 (18). This variation might be due to the difference in the time gap, cultural beliefs and the study population socio-demographic characteristics. The present study was conducted from the urban population, in which the habit of attending information and realizing it is higher in urban areas. Besides, our study was conducted in a specific area, whereas the result from Nigeria was from the national demographic health survey.

The current study found that maternal age greater than 35 years were two times more likely to have had higher decision-making autonomy compared to their counterparts. A similar result was reported from Southern Ethiopia, in which women greater than 30 years were more likely to be participated in the decisions on their own health (14). This might be due to as age gets increased their educational status may have also increased and respect between couples will be increased with age.

Adequate knowledge of neonatal danger sign plays a great role in the decision making autonomy of women on maternal and neonatal health. Thus, this study revealed that women who had adequate knowledge of neonatal dangers signs were two times more likely to have had higher decision-making autonomy compared to women who had poor knowledge of neonatal danger signs. This finding is consistent with a study conducted in Bale zone, Ethiopia, in which women

who had adequate knowledge of maternal and child health were independently associated with women’s autonomy on maternal and child healthcare utilization (1). This may be due to the fact that having good knowledge of maternal and neonatal health enforces women to challenge their husbands because they comprehend the seriousness of the illness.

Similarly, this study found that monthly income had a direct association with women’s decision-making autonomy on maternal and neonatal healthcare utilization. Women who had a monthly income of 5000 ETB and above were three times more likely to have had higher decision- making autonomy for their own health, neonatal health and other social and economic activities compared to women who had a monthly income of less than 1200 ETB. This finding is in agreement with a study conducted in Southern, Ethiopia (14). This could be explained by economically capable women are more likely to use communication devices (like television, radio and magazine) and utilize maternal and neonatal health information than the poor population.

Moreover, women who got husband’s involved in MNCH related activities were 2.36 times more likely to have had higher decision-making autonomy compared to women who hadn’t get husband involvement. Studies elsewhere reported that husband involvement encourage women’s participation in social and economic domains (23), associated with women’s use of skilled maternal and neonatal health services (24), better intra-spousal communication, birth preparedness and readiness for complications and utilization of maternal health services which in turn improve women’s decision-making autonomy and shared decision between couples (11,25). The other explanation might be the certainty of the women will be increased because of being helped and encouraged by their husbands and accordingly increased shared decision.

Conclusion

Women's decision-making autonomy on maternal and neonatal healthcare utilization was optimal. Older maternal age, higher economic status, adequate knowledge of neonatal danger signs and husband involvement in MNCH were the factors that contribute women to have had higher decision-making autonomy on maternal and neonatal healthcare utilization. There is a need for integrated work to increase women's decision-making autonomy thereby increasing utilization of maternal and neonatal healthcare.

Limitation of the study

Social desirability bias may not be eliminated. However, a better way of understanding was created by trained data collectors about the importance of the study and their participation can play an incredible effect on the benefits of the study findings.

Abbreviations

AOR	Adjusted Odds Ratio
ANC	Antenatal Care
CI	Confidence Interval
COR	Crude Odds Ratio
EDHS	Ethiopian Demographic Health Survey
ETB	Ethiopian Birr
MNCH	Maternal, Neonatal and Child Health
NDS	Neonatal Danger Signs Okay
PNC	Postnatal Care
SDG	Sustainable Development Goal
SPSS	Statistical Package for Social Science

Acknowledgements

We would like to thank the University of Gondar for providing study ethical clearance and financial support. Our thankfulness also goes to all data collectors and study participants. We are glad to Debretabor Town Health Office for giving us a permission letter.

Authors' contributions

All the authors had significant involvement in the conception and designing the study, acquisition of data, analysis and interpretation of data, took part in drafting the article, revising the article, gave final approval of the version to be published, have agreed on the journal to which the article has been submitted and agree to be accountable for all aspects of the work.

Availability of data and material

The datasets collected and analyzed for the current study are available from the corresponding author and can be obtained on a reasonable request.

Disclosure

The authors declare that they have no conflict of interest for this work.

References

1. Nigatu D, Gebremariam A, Abera M, Setegn T, Deribe K. Factors associated with women ' s autonomy onmaternal and child health care utilization in Bale Zone : a community based cross-sectional study. 2014;
2. Jahan F, Hossain S, Mahmud KF. Factors influencing women' s decision making power: evidence from Bangladesh urban health survey data. 2015;3(3):133–50.
3. Pratley P. Associations between quantitative measures of women ' s empowerment and access to care and health status for mothers and their children : A systematic review of evidence from the deve ... Social Science & Medicine Associations between quantitative measure. Soc Sci Med. 2018;169(August 2016):119–31.
4. Hou X, Ma N. The effect of women ' s decision-making power on maternal health services uptake : evidence from Pakistan. 2013;5(April 2012):176–84.
5. Trends in maternal mortality 2000 to 2017: estimates by WHO, UNICEF, UNFPA, World Bank Group and the United Nations Population Division. Geneva: World Health Organization; 2019: Licence: CC BY-NC-SA 3.0 IGO.;
6. UNICEF, WHO, World Bank and U-DPD. Levels & Trends in Estimates Developed by the UN Inter-agency Group for Child Mortality Estimation United Nations Levels & Trends in Child Mortality. 2017;
7. Asweto CO, Aluoch JR, Obonyo CO, Ouma JO. Maternal Autonomy , Distance to Health Care Facility and ANC Attendance : Findings from Madiany Division of Siaya County , Kenya. 2014;2(4):153–8.
8. Kariman N, Simbar M, Ahmadi F, Vedadhir AA. Socioeconomic and Emotional Predictors of Decision Making for Timing Motherhood Among Iranian Women in 2013. 2014;16(2).
9. Skakoon-sparling S, Cramer KM, Shuper PA. The Impact of Sexual Arousal on Sexual Risk-Taking and Decision-Making in Men and Women. Arch Sex Behav. 2015;
10. Personal M, Archive R. Munich Personal RePEc Archive Income Generation and Intra-Household Decision Making : A Gender Analysis for Nigeria Income Generation and Intra-Household Decision Making : A Gender Analysis for Nigeria. 2011;(27738).
11. Osamor P, Grady C. decision-making autonomy : empirical evidence from nigeria. 2018;50(1):70–85.
12. Tunku U, Rahman A. Factors Effect on Women Autonomy and Decision-Making Power

within the Household in Rural Communities Factors Effect on Women Autonomy and Decision-Making Power within the Household. 2014;(January 2011).

13. Jemberia MM, Berhe ET, Mirkena HB, Gishen DM, Tegegne AE, Reta MA. Low level of knowledge about neonatal danger signs and its associated factors among postnatal mothers attending at Woldia general hospital, Ethiopia. Matern Heal Neonatol Perinatol. 2018;4(1):5.
14. Press D. Health care decision making autonomy of women from rural districts of Southern Ethiopia : a community based cross-sectional study. 2017;213–21.
15. Ethiopian mini demographic and health survey.Addis Ababa, Ethiopia, and Rockville, Maryland UC and I, editor. Ethiopian mini demographic health survey. Ethiopian public health institute; 2019.
16. Centeral statical agency(CSA) (Ethiopia) and ICF.2016.Ethiopian demographic and health survey.Addis Ababa, Ethiopia, and Rockville, Maryland UC and I. No Title.
17. Akuma AO. Woman ' s autonomy on maternal health service utilization and associated factors in Ambo town , west showa zone , Ethiopia. 2019;(November).
18. Wado YD. Women's autonomy and reproductive health-care-seeking behavior in Ethiopia. Women Heal. 2018;58(7):729–43.
19. Transforming our world: the 2030 agenda for sustainable development. United nations. 2016;
20. Nigatu SG, Worku AG, Dadi AF. Level of mother's knowledge about neonatal danger signs and associated factors in North West of Ethiopia: A community based study. BMC Res Notes. 2015;8(1):4–9.
21. Anwar-ul-Haq, Durrani HM, Kumar R, Durrani SM. Recognizing the Danger Signs and Health Seeking Behaviour of Mothers in Childhood Illness in Karachi, Pakistan. Univers J Public Heal. 2015;3(2):49–54.
22. Ghose B, Feng D, Tang S, Yaya S, He Z, Udenigwe O, et al. Women ' s decision-making autonomy and utilisation of maternal healthcare services : results from the Bangladesh Demographic and Health Survey. 2017;1–8.
23. Jennings L, Na M, Cherewick M, Hindin M, Mullany B, Ahmed S. Women ' s empowerment and male involvement in antenatal care : analyses of Demographic and Health Surveys (DHS) in selected African countries. 2014;1–11.

24. Rahman AE, Perkins J, Islam S, Siddique AB, Anwar MR, Mazumder T, et al. Knowledge and involvement of husbands in maternal and newborn health in rural Bangladesh. 2018;1–12.

25. Pandey KK, Singh RD. Womens Status , Household Structure and the Utilization of Maternal Health Services in Haryana (India). 2017;10(1):1–10.

List of tables There are a lot of frequency values that are conflicting in your computation and as reported
Your cross-tabulation using multivariate conflicts with the frequency values

Table1: Socio-demographic characteristics of study participant in Debretabor, Northwest Ethiopia, 2019 (n=730)

Charactestics	Frequency	Percentage (%)
**Age of women in year**		
18-24	112	15.3
25-34	418	57.3
≥35	200	527.4
**Religion**		
Orthodox Christian	708	97
Muslim	13	1.5
Protestant	4	0.7
**Educational status of the women**		
No formal education	80	11
Primary education	131	17.9
Secondary education	160	21.9
Diploma and above	359	49.2
**Occupation of the women**		
House wife	371	50.8
Government employee	217	29.7
Self employed	26	3.6
Merchant	87	11.9
Others[a]	29	4
**Husband educational status**		
No formal education	24	3.3
Primary	63	8.6
Secondary	156	21.4
Diploma and above	487	66.7

**Husband occupation**		
Government employee	406	55.6
Merchant	155	21.2
Self employed	115	15.8
Daily labor	45	6.2
Others [b]	9	1.2
**Exposure to media**		
Yes	639	87.5
No	91	12.5
**Family size**		
<3	155	21.2
3-5	533	73
>5	43	5.8
**Average monthly income of the family**		
<1200 ETB	3	4.5
1201-3000 ETB	242	33.2
3001-5000 ETB	246	33.7
>5000 ETB	209	28.6

a= student and daily labour, b= student and carpenter

Table2: Reproductive history ad maternity healthcare service-related characteristics of study participant in Debretabor, Northwest Ethiopia, 2019 (n=730)

Characteristics	Frequency	Percentage (%)
**Parity**		
1	197	27
2-4	483	68.2
>4	50	6.8
**Had ANC**		
Yes	712	97.5
No	18	2.5
**Number of ANC follow up (n=712)**		
<4	288	40.4
≥4	424	59.6
**Place of delivery**		
Government hospital	586	80.3
Health center	99	13.6
Private hospital/clinic	11	1.5
At home	34	4.7
**Birth assistant**		
Health professionals	697	95.5
TBAs	33	4.5
**Had PNC**		
Yes	686	94
No	44	6
**Number of PNC (n=686)**		
<3	279	40.7
≥3	407	59.3
**Husband involvement**		
Involved	338	46.3
Not involved	392	53.7
**Visited by HEWs**		

Yes	388	53.2
No	342	46.8
**History of neonatal death**		
Yes	25	3.4
No	705	96.6
**Time taken to the health facility**		
<30 minute	717	98.2
≥ 30 minute	13	1.8

ANC= antenatal care, HEWs= health extension workers, PNC = postnatal care

Table 3: Women's household decision-making autonomy characteristics, in Debretabor, northwest Ethiopia, 2019**.**

Variables	Frequency	Percentage (%)
**Decision on her own health**		
Self	61	8.4
Jointly	526	72.1
Husband	143	19.6
**Decision on large household purchase**		
Self	15	2.1
Jointly	563	77.1
Husband	152	20.8
**Decision on small household purchase**		
Self	156	21.4
Jointly	545	74.7
Husband	29	4
**Decision on children and/ or neonatal health**		
Self	37	5.1
Jointly	619	84.8
Husband	74	10.1
**Decision on to have additional child**		
Self	27	3.7
Jointly	625	85.6
Husband	78	10.7
**Decision on visiting to family and/ or relatives**		
Self	113	15.5
Jointly	546	74.8
Husband	71	9.7
**Decision on husband's earning**		
Self	15	2.1
Jointly	496	67.9
Husband	219	30

**Decision on cooking daily foods**			
Self	498	68.2	
Jointly	202	27.7	
Husband	30	4.1	
**Decision to utilize maternal health services**	Was this the only subset of decision making proxies that was used as your outcome? If not, this is entirely what I could perceive was your outcome variable in this study		
Self			
Jointly	77	84.9	Check theses percentages
Husband	620	10.5	
	33	4.5	

Table 4: Bivariable and multivariable logistic regression analysis of factors associated with women's decision-making autonomy among women who had an infant age of one year and below in Debretabor, Northwest Ethiopia, 2019 (n=730).

You have not used this term to describe your outcome of interest till now. Is this a sub-sample of your outcome of interest?

Variables	**Women's autonomy**		COR (95%CI)	AOR (95%CI)
	Higher decision-making autonomy	Lower decision-making autonomy		
**Age of women**				
18-24	70	42	1	1
25-34	318	100	1.9 (1.22, 2.97)	1.59(0.98, 2.56)
≥ 35	160	40	2.4 (1.43, 4.02)	2.08 (1.19, 3.62)*
**Women educational status**				
No formal education	56	24	1	1
Primary education	91	40	0.98(0.53, 1.78)	0.99 (0.5, 1.89)
Secondary education	111	49	0.97(0.54, 1.74)	0.94 (0.5, 1.87)
Diploma and above	290	69	1.8 (1.04, 3.11)	0.85 (0.45, 1.64)
**Husband involvement**				
Involved	287	51	2.82 (1.96, 5.7)	2.36 (1.55, 3.43)**
Not involved	261	131	1	1
**Knowledge of NDS**				
Knowledgeable	234	41	2.56(1.74, 3.77)	2.11(1.4, 3.2)**
Not knowledgeable	314	141	1	1
**Media exposure**				
Exposed	493	146	2.21 (1.4, 3.5)	0.99 (0.57, 1.74)
Not exposed	55	36	1	
**Parity**				
1	135	62	1	1
2-4	373	110	1.56(1.08, 2.25)	1.23 (0.77, 1.95)
>4	40	10	1.84(0.87, 3.91)	1.13 (0.46, 2.77)

**Income**				
1200 ETB < 1200 ETB	16	17	1	
1201-3000 ETB	173	69	2.66(1,27, 5.57)	2.68 (1.23, 5.57) *
3001-5000 ETB	187	59	3.37(1.6, 7.1)	2.37(1.07, 5.22) *
>5000 ETB	172	37	4.9 (2.3, 10.6)	3.1(1.36, 7.07)*

* P value <0.001, ** p value <0.05, 1 reference category, ETB = Ethiopian Birr, NDS = Neonatal danger signs
