# Supplementary material for: Married women’s decision-making autonomy in the household and maternal and neonatal healthcare utilization and associated factors in Debretabor, northwest Ethiopia
Source: PLoS One. 2021 Sep 27;16(9):e0255021. doi: 10.1371/journal.pone.0255021 (PMC8476028; doi:10.1371/journal.pone.0255021)
Supplement: Attachment — Submitted filename: Response to Reviewers.docx [file pone.0255021.s004.docx]

**May 30, 2021**

**Manuscript PONE-D-20-25863**

**Response to Reviewers**

First of all, we would like to acknowledge your constructive comments on and valuable improvements to our paper. We have incorporated the suggestions and comments with tracked changes in the revised manuscript and a point-by-point response, please see below.

**Response to Editor Comments**

| S.N | Comments | Responses |
| --- | --- | --- |
| 01 | Please ensure that your manuscript meets PLOS ONE's style requirements, including those for file naming. The PLOS ONE style templates. | Thank you very much for your genuine advice and we have revised the manuscript as per your kind recommendation; PLOS ONE style template. |
| 02 | . Please include additional information regarding the survey or questionnaire used in the study and ensure that you have provided sufficient details that others could replicate the analyses. For instance, if you developed a questionnaire as part of this study and it is not under a copyright more restrictive than CC-BY, please include a copy, in both the original language and English, as Supporting Information. If the original language is written in non-Latin characters, for example Amharic, Chinese, or Korean, please use a file format that ensures these characters are visible. | We think this is an excellent suggestion. We have submitted the questionnaire as ‘‘supporting information’’ per your guidance. Thank you in advance. |
| 03 | Please state whether you validated the questionnaire prior to testing on study participants. Please provide details regarding the validation group within the methods section. | The questionnaire for this study was adapted by reviewing literature and cited in the revised manuscript. Thereafter, the content validity of the study tool was assessed by a group of senior researchers (1 person from the department of Reproductive and child health, 2 assistant professors (PHD candidate) from the department of Clinical Midwifery, and 1 assistant professor (PHD candidate) from the department of Women’s and family health) to check the suitability of the questionnaire. Besides, pretest was done on 5% of the calculated sample size before the actual data collection outside the study site. Detail description is written in the revised manuscript |
| 04 | Thank you for stating the following in the Acknowledgments Section of your manuscript:  [We would like to thank the University of Gondar for providing study ethical clearance and financial support.]  We note that you have provided funding information that is not currently declared in your Funding Statement. However, funding information should not appear in the Acknowledgments section or other areas of your manuscript. We will only publish funding information present in the Funding Statement section of the online submission form.  Please remove any funding-related text from the manuscript and let us know how you would like to update your Funding Statement. Currently, your Funding Statement reads as follows:  [No funding. We are from a low income country, Ethiopia.]  Please include your amended statements within your cover letter; we will change the online submission form on your behalf. | We really appreciate you for your kind constructive feedback, no any specific fund was gain for this study. We have described in the cover letter as per your kind recommendation. |

**Response to Reviewer(s) comments**

**Reviewer #1**

| S.N | Comments | Responses |
| --- | --- | --- |
| 01 | The literature review is inadequate and not properly organized. | Thank you, we have accepted and corrected the comment in the revised manuscript. |
| 02 | The statistical analysis is not rigorous and no regression model is explicitly specified. | Thank you for your considerate comment, we tried to explain the statistical analysis and the regression model we used explicitly in the revised manuscript |
| 03 | Results are not reported and explained clearly. | Thank you very much, accepted and corrected in the revised manuscript. |
| 04 | The manuscript is written in poor English | Thank you for the comment, the language was reviewed exhaustively.  It is also clearly visualized via track changes in the revised manuscript. |

**Reviewer #2**

| 01 | Overall, the paper provides important evidence regarding the decision-making process on the utilization of maternal and neonatal health services. I do, however, have comments for some parts of the paper.  In many parts of the world, parents and other family members, especially mothers or grandmother play an important role in women's decision making on healthcare use. Authors should clarify why other family members were not included in the response of the decision-making variable. | Thank you for encouraging comments.  The likely replies for each question regarding the decision making process were:   1. women alone, 2. together with her husband, 3. husband only or 4. Other specify   However, after the actual data collection, there was no response for the ‘’other’’ option. Thus, in this study, the responses were me alone, together with my husband, or husband alone. Please see the tool. |
| --- | --- | --- |
| 02 | A number of independent variables (e.g. time taken to health facility, visited by HEWs and others) which were reported in the table should also be described in the methods section. | You have pointed out an important issue. We have done, please see in the ‘’measurement’’ section. |
| 03 | It is important to present the proportion of healthcare practices and knowledge among autonomous women in table or figure. | Thank you, we have been present the proportion by figure. |
| 04 | Study found that women with adequate knowledge of neonatal dangers signs tend to have higher decision-making autonomy compared to those with poor knowledge. However, the proportion of autonomous women with good knowledge of neonatal danger signs was only 32%. This should be clarified. | Thank you very much for your suggestion. However, we didn’t mean that the figure 32% as adequate knowledge rather ‘’Of the autonomous study participants only 32% had adequate knowledge about neonatal danger signs. And the other finding; women with adequate knowledge of neonatal dangers signs tend to have higher decision-making autonomy compared to those with poor knowledge. We hope the reviewer and the editor will understand the concern. If you have any suggestion regarding this issue, we will be happy to receive it kindly. |
| 05 | Husband involvement is one factor associated with women decision-making autonomy. Should prenatal care also target husband to increase their awareness and support to mothers? It would be good to suggest more practical recommendations on how to improve the decision-making process within the household. | *Thank* you for your kind recommendation, yes it would be nice if awareness creation for husbands should be given during the prenatal period and we incorporated the recommendation. |

**Reviewer #3**

**(Dr. Phillips Obasohan, PhD.)**

| 01 | The study is not clear whether MNHC is the only aspect of women autonomy that was considered? | Thank you dear reviewer for insightful and careful reading,  No, MNHC is not the only aspect of women autonomy; the entire aim of this study was to assess married women’s autonomy in the household and on maternal and neonatal healthcare utilization and associated factors in Debretabor, northwest Ethiopia. Since maternal and neonatal health the top priority problem worldwide, the topic inclined to “married women’s autonomy on maternal and neonatal healthcare utilization and associated factors in Debretabor, northwest Ethiopia”. So, we are pleased to do little modification to the topic (married women’s autonomy in the household and on maternal and neonatal healthcare utilization and associated factors in Debretabor, northwest Ethiopia) and we amend the whole text as per your kind concern. Thank you in advance. The corrections are clearly visualized via track change throughout the revised manuscript. |
| --- | --- | --- |
| 02 | Are these items not proxies women autonomy in this study? it is for this sentence in the abstract section (Besides, the proportion of at least four antenatal visits, delivery at a health facility, postnatal checkup, knowledge of neonatal danger signs and appropriate health-seeking practices for sick newborns among autonomous women were 52.1%, 56.1%, 71.4%, 32%, and 80% respectively. | Thank you, it was simply to describe the proportion of these variables among autonomous women. The ultimate aim of the study was to assess women’s autonomy and associated factors in the household and on maternal and neonatal healthcare utilization. In the meantime, we describe how much percent of women had good proportion of the aforementioned items. |
| 03 | State also how many are married, since that group is the unit of analysis if the data is available from your source (in the method section) | Thank you, all study participants were married. Stated as ‘’A community-based cross-sectional study was conducted from October 1st to 30th, 2019. A two-stage sampling technique was used to select **730** married women’’. Please kindly see the text in the ‘’methods’’ section. |
| 04 | What is a response variable? This is confusing? | Thank you, it was to mean ‘’response’’ only. We re-write it in the revised manuscript. |
| 05 | Justify the inclusion of these as components of MNHC (Large household purchases and consumptions (7) Intra-household resource allocation and purchases (8) Husband’s earning and (9) Cooking daily foods) | Thank you for your insightful review, these variables are related to women’s decision-making autonomy in the household. We already made little modification on the topic as per your kind concern and amend the main text carefully. |
| 06 | How about a situation where neither the husband nor the woman make the decision on? I wonder that some of your respondents may fall into such category | Thank you, very thoughtful and incredible way of looking. You are right, initially, we have prepared the questionnaire based on your way of looking in which the women were asked “who in your household decides to use maternal health services and other household activities”. We expect a response of  1) me alone,  2) my husband alone  3) both of us  4) others specify.  Unfortunately, no response was for the other specify option. This might be due to the present study was conducted in the urban setting in which urban residences tend to be educated and being aware of the need for independent or joint decision for every household activity including healthcare service utilization as compared to women from rural area. The questionnaire is employed for data collection is attached as supporting information. |
| 07 | What are the independent variables utilized in this analysis? | Thank you, comment accepted and incorporated in the revised manuscript in the “measurement section”. |
| 08 | What is NDS? | Thank you, NDS=neonatal danger sign (described in the text at its first appearance and in the list of abbreviation). |
| 09 | From the measurement variable definition, what constitute overall DMA? | Thank you, nine questions were prepared related to women’s decision-making autonomy which constitute the overall women’s DMA. Stated as follows “who decides in your household (1) Healthcare for yourself (2), Healthcare for newborns and/ or children (3) Visit of family or relatives (4) To have additional children (5) Utilization of maternal and child healthcare services like ANC, PNC, and immunization (6) Large household purchases and consumptions (7) Intra-household resource allocation and purchases (8) Husband’s earning and (9) Cooking daily foods. Thus, the women’s response was coded into (2 = for women who were able to decide individually, 1= for women who were able to decide together with her husband and 0 = otherwise). The cumulative score was ranged from zero to 27. Accordingly, women who scored above the mean were considered highly autonomous whereas women who scored below the mean were less autonomous. The likely replies for each question were women alone, together with her husband, husband only or other. |
| 10 | How this was variable derived and scored from your questionnaire? | **Thank you very much, it was typing error. It was to mean maternal, neonatal and child healthcare utilization among autonomous women including having adequate ANC, institutional delivery, having PNC, and appropriate healthcare seeking practice for neonatal danger signs.** |
| 11 | I think you should be more specific about what you mean autonomous women: do you mean ' high autonomous women?' | Thank you, it was to mean higher autonomy and corrected in the revised manuscript |
| 12 | This is one of your two main objectives. Did you attempt to investigate the significance of these proportion, rather include their respective confidence intervals, since this is a survey analysis of sample? | Thank you, we didn’t attempt to check the significant association of decision-making autonomy and healthcare services. Rather we assess the proportion and associated factors of women’s autonomy. The association between women’s autonomy and maternal health services are search intensively. However, little was searched what factors deter the women’s decision making autonomy. |
| 13 | Which category of age group is referred to here? | Thank you, it was to mean the reference group (18-24) |
| 14 | 1. This can be a more acceptable term for the success in outcome (higher decision-making autonomy)? | Yes it was to mean higher decision-making autonomy, we accepted and revised thoroughly |
| 15 | There are a lot of frequency values that are conflicting in your computation and as reported your cross-tabulation using multivariate conflicts with the frequency values? | Thank you for deep review and careful reading for each point. We revised the whole table thoroughly. |
| 16 | Was this the only subset of decision making proxies that was used as your outcome? If not, this is entirely what I could perceive was your outcome variable in this study? | Thank you, we described earlier and we did little modification on the title and the whole manuscript accordingly. |
| 17 | Check the percentage (table 3 ) | Thank you once again in pointing out the errors, we corrected it in the revised manuscript. |
| 18 | You mentioned that your study differs from previous study because you added MNHU to the already known proxies for women autonomy index. Now, is this analysis based on just an aspect of the components of the proxies for women autonomy you itemized in ' measurement variable' section**?** | Thank you, our study was aimed to assess the proportion of decision-making autonomy and associated factors. So, we did little modification to the title and across the document. |
| 19 | You have not used this term to describe your outcome of interest till now. Is this a sub-sample of your outcome of interest? (The title of table 4) | Thank you, accepted and corrected in the revised manuscript |
